# Endoscopic submucosal dissection for a large cecal adenoma covering an appendiceal orifice after appendectomy

**DOI:** 10.1055/a-2218-2780

**Published:** 2024-01-09

**Authors:** Yuya Asada, Takashi Kanesaka, Tomoki Michida, Hidetoshi Satomi, Keiichiro Honma, Ryu Ishihara

**Affiliations:** 1 53312 Department of Gastrointestinal Oncology, Osaka International Cancer Institute, Osaka, Japan; 2 Department of Gastroenterology and Hepatology, Osaka University Graduate School of Medicine, Suita, Japan; 3 53312 Department of Diagnostic Pathology and Cytology, Osaka International Cancer Institute, Osaka, Japan; 4 Department of Gastrointestinal Oncology, Osaka International Cancer Institute, Osaka, Japan

Endoscopic submucosal dissection (ESD) is recommended for colorectal tumors that cannot be completely removed by snare-based techniques. Endoscopic resection for appendiceal orifice lesions (Toyonaga’s type 3: lesion entirely covers the orifice) is technically challenging because the appendiceal portion cannot be incised accurately 1 . Such lesions can be endoscopically removed if the patient has undergone an appendectomy, but submucosal fibrosis is a potential issue. Several reports have shown that ESD using a traction device or underwater strategies are useful for such lesions 2 3 4 . We demonstrate ESD for a lesion using the water pressure method ( Video 1 ), which is a technique that uses the waterjet function of an endoscope to secure the working space 5 .

Endoscopic submucosal dissection using the water pressure method for a large cecal adenoma covering the appendiceal orifice after appendectomy.Video 1

A 62-year-old woman, who had undergone appendectomy for acute appendicitis 45 years previously, was diagnosed with a 35-mm laterally spreading tumor in the cecum on colonoscopy ( Fig. 1 ). The scar of the appendiceal orifice could not be identified because the lesion was covering it. We performed ESD for the lesion with a FlushKnife BT-S (1.5 mm, DK2620J; Fujifilm Medical, Tokyo, Japan).

**Fig. 1:**
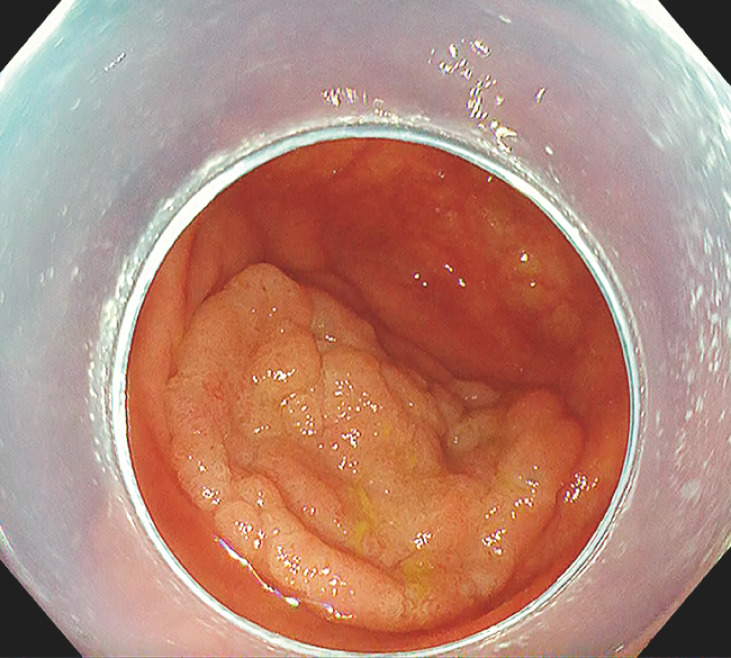
A 35-mm laterally spreading tumor covering the appendiceal orifice after appendectomy.

A solution of sodium alginate was injected into the submucosa for thickening. The endoscope faced the lesion perpendicularly, and endoscope stability was poor due to respiratory fluctuations. To secure a stable field of view, the water pressure method was used while sucking air from the cecum. Severe fibrosis was partly visible in the submucosa during ESD ( Fig. 2 ). En bloc resection was achieved while maintaining a good visual field using the water pressure method ( Fig. 3 , Fig. 4 ). The procedure was completed without any adverse events.

**Fig. 2:**
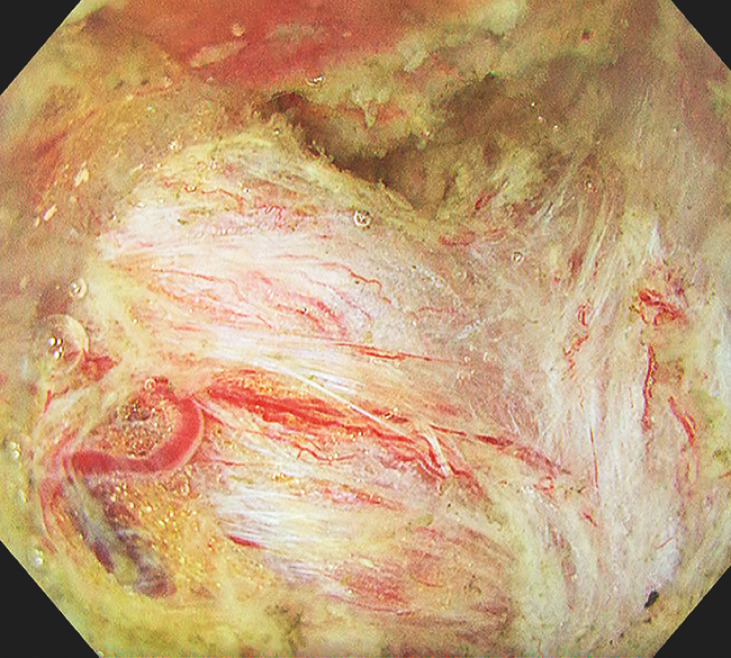
Severe fibrosis was partly visible in the submucosa during submucosal dissection.

**Fig. 3:**
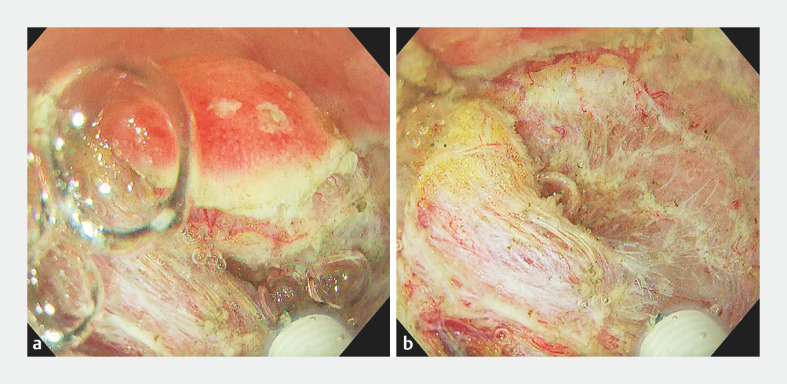
The water pressure method. **a** Before water injection. **b** Just after water injection.

**Fig. 4:**
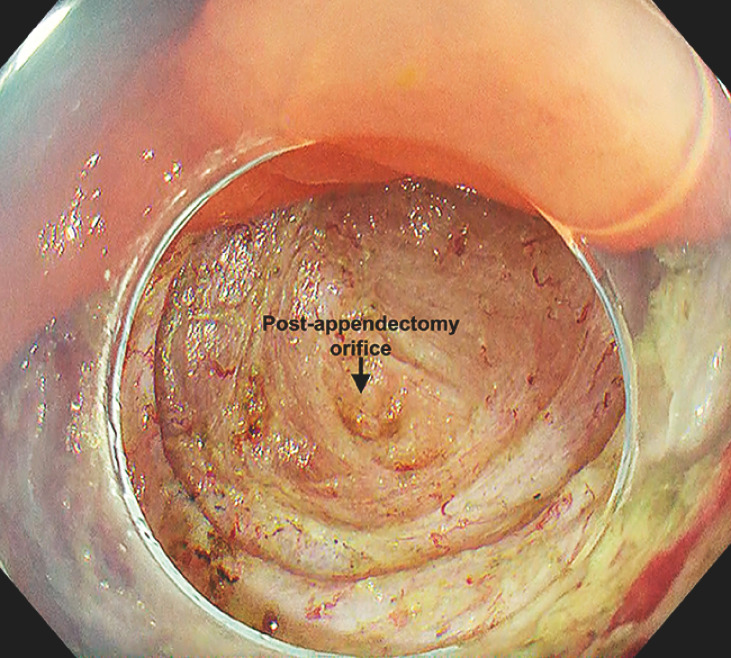
The appendiceal orifice was identifiable in the resection wound.

The patient was discharged on postoperative day 4. Histopathological examination revealed a high grade tubulovillous adenoma with negative margins.

A large cecal adenoma covering the appendiceal orifice after appendectomy could be removed by ESD with the water pressure method.

Endoscopy_UCTN_Code_TTT_1AQ_2AD

## Notes

Endoscopy E-Videos https://eref.thieme.de/e-videos
*E-Videos* is an open access online section of the journal *Endoscopy* , reporting on interesting cases and new techniques in gastroenterological endoscopy. All papers include a high-quality video and are published with a Creative Commons CC-BY license. Endoscopy E-Videos qualify for HINARI discounts and waivers and eligibility is automatically checked during the submission process. We grant 100% waivers to articles whose corresponding authors are based in Group A countries and 50% waivers to those who are based in Group B countries as classified by Research4Life (see: https://www.research4life.org/access/eligibility/ ). This section has its own submission website at https://mc.manuscriptcentral.com/e-videos .
